# Fourth year intellectual disability student nurses’ journey and future work intention: a qualitative study

**DOI:** 10.1186/s12912-022-01007-9

**Published:** 2022-08-08

**Authors:** Owen Doody, Pauline Meskell, Sylvia Murphy-Tighe, Maria Noonan, Liz Kingston

**Affiliations:** Department of Nursing and Midwifery, Health Research Institute, University of Limerick, Limerick, Ireland

**Keywords:** Intellectual disability, Workforce, Retention, Nurse, Student, Ireland

## Abstract

**Background:**

The aim of this qualitative study is to explore the views and experiences of final year BSc intellectual disability nursing students’ journey, future work plans and examine factors influencing their migration intentions following graduation.

**Methods:**

A qualitative component of a mixed methods study where a focus group interview was conducted with final year BSc intellectual disability nursing students (*n* = 10) from one University in Ireland in June 2019. A topic guide was utilised, and participant’s were interviewed about their programme, future work plans and migration intentions. An inductive approach was utilised, and data were analysed using a pre-existing framework for initial coding and thematic development. Duffy’s conceptual model of identity transformation provided a structure to analyse the data and map themes onto the conceptual framework.

**Results:**

The findings were mapped onto the five stages of Duffy’s (2013) conceptual model of identity transformation: Pre-Entry; Reaffirming; Surmounting; Stabilising and Actualising. Findings indicate that further work is required to promote intellectual disability nursing and address professional esteem issues, support for education and professional development, such as providing career guidance opportunities prior to course completion, development of clinical skills within their education programme and support for the professional development of new graduates. Participant’s identified uncertainty about career opportunities and saw scope for future professional development opportunities particularly in community-based work.

**Conclusion:**

This study has identified that final year intellectual disability nursing students are uncertain about career options and opportunities for intellectual disability nurses in other country’s. There is an urgent need for the intellectual disability nursing profession to articulate their practice and advocate for their role and contribution to the care of people with intellectual disability. This study identified a clear need for direction and information regarding intellectual disability nursing roles and career opportunities.

## Background

To provide high quality safe healthcare services, there is a need for sufficient appropriately trained staff to be in place at national, regional and local levels [1]. However, global health workforce requirements are expected to increase in the coming years due to population growth, demographic and epidemiological factors [1, 2]. In this context, the World Health Organisation (WHO) predicts a global deficit of 18 million skilled health workers by 2030 [2]. Many country’s are already experiencing challenges in the recruitment and retention of nurses, and this poses a significant strategic risk to the effective operation of a country’s health system in the coming years [3, 4]. In addressing these challenges each country’s national workforce planning strategic framework should encompass the WHO’s global code of practice on the international recruitment of health personnel principles [5]. Therefore, country’s need to proactively plan and manage their health workforce demand and supply as far as possible. Part of this strategic planning process involves managing the migration of nurses and this must be balanced against healthcare professionals’ freedom to pursue work and the need to limit excessive losses to a profession or workforce.

Registered nurses in intellectual/learning disability provide health, wellbeing and social care supports to individuals with an intellectual disability [6]. Ireland and the United Kingdom (UK) are the only two country’s specifically providing direct entry training programmes for intellectual/learning disability nurses [7], and therefore it may be anticipated that there would not be a retention or workforce issue. However, other factors such as staff stress and burnout, organisation culture, training and supervision [8] affect the retention of this specific discipline of nurses. Ireland and the UK have highly regarded nursing educational standards and given that English is the first language of students on these courses, there is a high international demand for graduate nurses from these jurisdictions and consequently a high level of opportunity for mobility [9].

Within published literature the definition of the term intellectual disability and an individual’s understanding of the label can be diverse [10]. This diversity is reinforced by the fact that the term learning disability is used in the UK and other terms are used interchangeably such as developmental disability, intellectual developmental disorder, disorders of intellectual development, and intellectual and developmental disability. However, intellectual disability is the term of choice internationally and the disparity in the use of these labels poses challenges when comparing international research. Intellectual disability does not constitute a homogeneous group, however in terms of diagnosis and classification there are several features which have gained widespread acceptance irrespective of the terminology or definitions used. The three core criteria are: significant impairment of intellectual functioning; significant impairment of adaptive/social functioning and onset before adulthood.

International evidence recognises that people with intellectual disability have poorer health [11], greater health comorbidities [12], present more often for treatment [13], have more complex needs [14], experience increased polypharmacy [15] and die earlier [16] compared to the general population. Furthermore, healthcare services have been identified as ill-equipped to respond to the needs of a person with intellectual disability. There is evidence that people with intellectual disability receive poorer quality care [17], have more preventable illness [18], experience greater health disparities [19] and untreated health needs [20]. This evidence highlights the need for an educated workforce with specific skills and training in intellectual disability. Intellectual disability nurses in Ireland and learning disability nurses in the UK have as a profession evolved over the last three decades in response to the changing landscape of service provision and in response to the complex healthcare needs of people with intellectual disability [21]. Intellectual disability nurses possess a wide range of specialised skills to meet the varied health, behavioural, advocacy, and societal needs of persons with intellectual disability [22]. Intellectual disability nurses meet the multifaceted healthcare needs of the person with intellectual disability through providing comprehensive care delivery and management that is appropriate to the individual through their specialised intellectual disability knowledge and skill [23]. Intellectual disability nurses adapt their nursing skills for chronic health conditions experienced among people with intellectual disability including pneumonia, aspiration, dehydration, constipation, seizures, motor deficits, allergies, otitis media, gastroesophageal reflux disease, diabetes, dysmenorrhea, sleep disturbances, thyroid disorders, behavioural/mental health issues, vision and hearing impairments, and oral health problems [24].Intellectual disability nursing has adapted, reshaped and refocused the profession over the past three decades to respond to social, cultural, legal and moral drivers. Their role has often been questioned and often poorly understood and this stems from a move to a more social model and person-centred planning ideology [25]. This role ambiguity has arisen as the social model, which underpins intellectual disability nursing, is perceived as the absence of nursing. Where in fact the social model within a healthcare context for people with intellectual disability is essential given their complex health needs, disparity in health and difficulties accessing mainstream healthcare. Consequently, the social model guides students to consider intellectual disability nursing within a wider society of supports, to challenge negative attitudes and prejudices that may exist and to consider the action required to address inequalities in health and to develop strategies to identify and support the person.

Although models of service provision for people with intellectual disability have changed and moved to more personalised services, there remains a requirement for an appropriately trained and skilled workforce [26]. However, internationally the focus has shifted from specialised nurses to untrained support staff [27]. The ongoing changes in service provision, questioning of need for a distinct intellectual disability nursing profession, along with the fact that intellectual disability services are marginalised and receive less research funding [10] often leads to internal and external questioning of the role of the intellectual disability nurse. This is evident within the UK where despite governmental commitment to the future of the learning disability nurse [28, 29], recruitment and retention issues exist with reducing numbers of learning disability nurses working in the National Health Service (NHS) [30, 31]. Recruitment and retention issues identified indicate anxiety about: the future of the profession [32], downgrading of the nursing workforce [33] and questioning the profession’s future as a separate field of nursing [34]. In addition, the significance of public perceptions of intellectual disability nursing is a consideration [35] and the influence of societal stereotyped views of what a nurse is and does [36]. The challenge of nursing staff turnover presents a significant barrier to building a specialised nursing workforce [37].

Given the fact that intellectual disability nurse training is only available in the UK and Ireland, the past and ongoing changes is service delivery models, the questioning of the intellectual disability nursing profession, and the multifaceted health and communication needs of persons with intellectual disability it is clear workforce planning needs to be a priority. Across nursing the shortage of nurses threaten the ability of health services to provide quality safe care [38, 39] and migration is a contributing factor to nursing/midwifery shortages [40]. Identification of workforce planning, and retention strategies are paramount [41] however, this requires an understanding of factors influencing nurses’ decision to migrate and or take up employment in their home country. The transition from student to registered nurse is identified as a significant time point as turnover is high particularly in the first year following qualification [37]. Staff turnover impacts on a healthcare organisation’s funding as it is expensive and time consuming to recruit new nurses into the workforce [37]. However most importantly staff turnover impacts on the provision of quality and safe person-centred care, patient morbidity and mortality rates [42]. Safe staffing levels are associated with improved outcomes including reduced medical and medication errors, decreased patient complications and mortality, improved patient satisfaction, reduced nurse fatigue and burnout and improved nurse retention and job satisfaction [43]. The issues of recruitment and retention for this specialised field of nursing is important as little is known from an Irish perspective on the factors affecting recruitment and retention of intellectual disability nurses. Furthermore, it is valuable to consider recruitment and retention prior to graduation as it gives an opportunity to consider interventions that may influence recruitment and retention at the point of new graduates entering a workforce as one strategy to secure a sustainable workforce for the future [37]. This paper aims to identify fourth year intellectual disability nursing students’ concept of intellectual disability nursing and employment intentions.

## Methods

### Study design

An explanatory concurrent mixed method research design was used to explore the migration intentions of fourth year graduating nursing and midwifery students in Ireland. The study was conducted in two phases and all data were collected between May and June 2019. Phase one of the study a survey of BSc nursing (general, mental health, intellectual disability) and BSc midwifery students is reported separately [9]. This paper reports phase two, a focus group interview with intellectual disability nursing students (*n* = 10) in one higher education institution in Ireland to explore their journey and explain and expand upon factors that influence on their future work and migration intentions. A participant information leaflet (PIL) outlining the nature, aims and purpose of the study and an invitation to participate in both phases of the study was emailed to all fourth-year students. Students were advised that participation in one phase did not require participation in the second phase. A date and time for the focus group was arranged outside of class time and prior to the focus group, participant’s were given an additional PIL and asked to sign a consent form.

### Study setting

The study was set in one higher education institution involved in nursing and midwifery education in Ireland.

### Ethical approval

Ethical approval for the study was obtained from Education and Health Science Research Ethics Committee (2019_05_08_EHS) and permission to access was obtained from the department head. Participant’s (*n* = 10) were informed that confidentiality was a shared responsibility among focus group participant’s and were asked to sign a declaration of confidentiality to ensure that the discussion and identity of fellow participant’s would not be disclosed outside the focus group. Participation was voluntary and participant’s were informed that they could withdraw from the study at any time.

### Sample

A total of ten students returned an expression of interest form indicating their willingness to participate in the focus groups and all participated in the study. The sample were all female ranging in age from 22 to 34 years. Three participant’s had accessed the programme via the mature entry route and the remainder were school leavers at the time of entry.

### Data collection

Focus groups were chosen for this phase of the research, after recruitment ten participant’s participated in one focus group which was conducted in a private room to facilitate a discussion among participant’s about their views and experiences around their intention following graduation, using a topic guide, developed from the literature and previous national survey [9]. Open questions and follow up probes were used. The focus group lasted approximately 50 min and were audio-recorded with consent.

### Data analysis

Transcripts were transcribed verbatim, anonymised, checked for accuracy and analysis performed utilising a form of ‘best fit’ framework analysis, where a pre-existing framework was used for initial coding and theme development. The framework utilised was based on Duffy’s conceptual model of identity transformation [44] which encompasses five stages: pre-entry, reaffirming, surmounting, stabilising and actualising. This analytic framework was chosen as it was developed through constructivist grounded theory techniques in an investigation of transition where participant’s conceptualised their personal and professional development identities over time. Utilising an inductive approach, data was coded and mapped onto the five stages of the conceptual framework by two authors (OD, LK) independently by reading and rereading the transcripts. The team met to discuss and confirm the mapping which is reported in the form of domain/theme, sub-theme/category and code/sub-category (Table 1) and supported by participant’s illustrative quotes in the results. Duffy’s conceptual model of identity transformation [44] provided a useful analytical structure to inform and shape an understanding of intellectual disability student nurses’ professional development and identity and migration intentions.

**Table 1:** Duffy’s (2013) Conceptual Model (Pre-Entry; Reaffirming; Surmounting; Stabilising and Actualising) and Theme Mapping

Theme	Pre-entry	**Reaffirming**	**Surmounting**	**Stabilizing**	**Actualizing**
Sub-theme	Choice Exposure Prior knowledge and understanding	Belonging	Placement Challenges and limitations	Developing	Unpredictability New future / opportunity Travel / mobility
Code	Need to choose something Not first choice Not first nursing choice Previous work experience Employed in sector as carer Social contact trough drama Advise others to have experience Previous experience and contact Information from school, friends doing course Went in blind Had to go into something	Short placements Varied placements Other settings Settling in Role Preferences Identity	Settings Community versus residential Residential setting Gaining confidence Recognition Clinical skills Learning on the job Assuming lead in own learning	Exposure and experience Learning from experience It's not black and white Care approach Additional interests Role clarity	Limited job opportunity Job security Complex landscape Need direction and information Service landscape Lead roles Care management and coordination Dual qualification Other healthcare professional training Travel desire Recognition Positive feedback from others Friends gone Perceived positive work environment Stay due to family Come back in a few years

## Results

Analysed data were mapped onto the five stages of Duffy’s conceptual model of identify transformation [43] and presented under the following themes:

### Pre-entry

The theme of pre-entry was evident within the analysis stage when mapping the coded data and the subthemes of choice, exposure, prior knowledge and understanding were evident. Participant’s discussed their choice to enter nursing and while half of the participant’s had previous exposure to intellectual disability nursing it was not their first course choice or first nursing course choice and this resulted in students changing or leaving the course. However, while it may not have been a first choice for some, participant’s reported adjusting to the course and growing to love it.

*“I just had to go into something …. I wanted the guards (Irish Police Force) but they hadn't recruited in a few years …. so I had to go into something, but it's been great, I love it …. three went to general and one left altogether”.*

For those with prior exposure this helped them choose intellectual disability nursing and settle into their programme. Experience ranged from social contact through a drama group, to exposure during school placements or work experience and employment as a carer.

*“I went to a unit within the service (names removed) …. we did a show with them in the (name removed) theatre …. I did get experience and I loved it …. I was care staff for a few years”.*

Some students did not have any prior experience of intellectual disability nursing prior to accepting a place on the course and this was identified as a reason why some students left the programme. Participant’s suggested that exposure and experience would be beneficial to assist prospective students to be informed of the course and consider intellectual disability nursing as a career choice.

*“Get experience first, cos how many people did we lose in the first year …. I'd say work as care staff for a while first”.*

Prior knowledge and understanding of intellectual disability nursing was seen as essential to retention of students on the course. Participant’s who stayed on the programme had obtained information about the programme prior to choosing the course through schools and friends.

*“I found out about it in school …. one of my friends was a year above me and she did this course, so she'd give me information about it”.*

However, some knew very little about the programme and articulated they were entering blind but there was a need to just choose something. Regardless for those who remained on the course they found it enjoyable.

*“I knew nothing about it until I went into it …. I went into it blind …. I just had to go into something, I didn't know what I was going into, but it's been a lovely course”.*

### Reaffirming

The theme of reaffirming was evident within the analysis stage when mapping the coded data. The coded data all mapped onto the subtheme of belonging and participant’s described ‘a sense of belonging’ developed through engagement with a variety of placements provided across intellectual disability and other nursing care settings during their programme. Such placements created a sense of settling in, however, while variety was seen as positive this was finely balanced by the fact that many placements were of a short duration.

*“We'd have done various placements but only a few weeks at the most in each …. you can be working in the community, in family homes, in residential care …. and we did weeks in general, paediatrics, mental health”.*

The variation offered in clinical placements sites assisted students to change their views on intellectual disability nursing as a career choice.

*“If I was asked that a couple of years ago, I would have said Acute setting was what I wanted but not now…. I'd rather community setting”.*

A key aspect of reaffirming was the development of participant’s identity as an intellectual disability nurse and part of this identity formation involved reconciling misconceptions of this minority discipline of nursing.

*“It is a level 8, you know that it is a degree, and we are nurses we do all the other things other nurses do”.*

Existing nurses had a key role in forming participant’s identity through engagement and mentorship. However, a negative aspect in identity formation were existing staff’s sense of value and commitment to their profession and their uncertainty led to participant’s questioning their value rather that consolidated it.

*“You walk in, and a nurse is asking you questions keeping you on your toes …. but if you're working with someone who is fed up with their own job, they're going to tell you to get out …. but the whole RNID degree is about holistic care, and you know it is a different attitude and that’s important”.*

### Surmounting

The theme of surmounting was evident within the analysis stage when mapping the coded data formed the subthemes of placement and challenges and limitations. Most of participant’s placements were in residential settings and participant’s were aware of the landscape shift of service to the community for people with intellectual disability and were surprised there were not more community placements.

*“The majority of it you could say that 80% of it has been in residential settings …. I'm surprised there isn't more placement community based …. but I've been in two community houses back-to-back, two months and two months”.*

While placements were predominantly in residential setting participant’s developed through learning on the job. However, a need for a greater focus on clinical skills development in the University setting was identified to enable participant’s gain greater confidence.

*“There’s hands on learning all the time when you’re on placement … and most of it has been in a residential setting and that's the only limit I think but clinical skills, we don't get enough clinical skills experience or teaching …. we only got an hour on injections, and we did that when in second year …. I know you're going to learn on the job as well but there needs to be more practical skills and OSCE’s …. you'd get it but not enough to be confident”.*

Key to surmounting challenges was assuming leadership in one’s own learning.

*“It's very hard sometimes to say can I do the medications with you because there's not enough people on the floor but once you just say it to them you do get more experience with medications, and they will let you help and let you get your experience with medications.*

### Stabilising

The theme of stabilising was evident within the analysis stage when mapping the coded data. The coded data all mapped onto the subtheme of developing where exposure to clinical practice and experience enabled participant’s create their identity and stabilise their understanding of their role. The changing landscape of service provision meant that students were exposed to a variety of care models with a holistic care approach evident across services.

*“We have the clinical, certain residential settings would be more clinical so you’re learning on the job you'd have like PEG experience …. we do all the other things that nurses do and it’s a holistic approach to like you know fulfilling somebody's life …. one community house is like the dream of community living and the other one can be all clinical or have extremely challenging behaviour and be very limited for the service users and it's opened my eyes to the congregated settings, it's not all black and white”.*

While participant’s programme of study and placement were rewarding, they also created additional interests for consideration of their future careers related to intellectual disability or nursing.

*“I'd love to do teaching, I'd love to teach kids with an intellectual disability or go to (named place) and do my children's nursing”.*

One issue of concern to participant’s was the aspect of role clarity which they had to address within themselves during their programme.

*“When we're on placement, and you're supposed to be getting nurse experience, but you do end up being the care staff, your kind of maybe on the floor while they're getting things done or doing their work”.*

### Actualising

The theme of actualising was evident within the analysis stage when mapping the coded data formed the subthemes of unpredictability, new future and opportunity, and travel and mobility. Given the uncertainty and direction from intellectual disability nursing leadership regarding the transition of intellectual disability nurses within the changing landscape of service provision participant’s articulated concerns regarding limited job opportunity and job security.

*“They have trained us for four years and there's not one job opportunity for us, at the moment anyway… It's kind of just a kick in the teeth then if they're not even offering one of us a job, you'd think they might have a few posts going …. a few years ago, they gave five jobs to one year but then they let them go again”.*

Participant’s expressed insecurity regarding the recognition of the intellectual disability nurse’s role internationally which may limit opportunities to secure employment in other country’s.

*“But intellectual disability nursing it’s not recognised in places like America is it”.*

Participant’s identified a clear need for direction and information regarding their role and career opportunities within Ireland.

*“We don't know where it's going, you have to work a little bit harder and figure it out, it would be helpful for someone to sit down and actually tell us the possibilities for our future like* *I imagine someone coming in and saying you can actually do this or you can do this, there should be always someone you can go to like a manager or someone senior to you so if you are not sure of something that you can go to them”.*

However, participant’s also saw opportunities in their nursing career pathway and role development.

*“I think as it moves out to community that there's going to be a need for community CNS's (clinical nurse specialists) and community CNM’s (clinical nurse managers) and we'll be like public health nurses but for intellectual disability …. CNS’s and ANP's (advanced nurse practitioners) that's where I hope we're going …. the RNID (registered nurse intellectual disability) is overseeing care, interventions are signed up and written up by the nurse and if someone needs to go and have their medication's reviewed then a nurse will step in or if something needs support the nurse will step in”.*

*“For me there is a need around nutrition and also palliative care, we will be more clinical as well as we move out to community …. out in the community not in a residential setting, I'm hoping that's the future”.*

To actualise their role fully some participant’s saw a need to further develop their nursing through a dual qualification. However, there was also a desire to do other healthcare professional training and leave nursing.

*“I would like have a dual qualification, I’d do my children's nursing, or I'd like to do occupational therapy or do speech and language”.*

However, many want to stay within intellectual disability services but would like to diversify and compliment their nursing role.

*“I'd like to still work in the intellectual disability sector, but just do something a bit different …. I'd like to do dietetics in a post grad, but that's for CNS, I still want to work in intellectual disability …. I'd love to teach kids with an intellectual disability”.*

Part of the actualising process was the realisation of the role, expectations of the role and the need to transition from student to staff.

*“You have to have to get at least six months work experience they expect so much when we qualify, the first six months as an actual staff nurse you will learn so much, we will be actually doing it yourself, being responsible for it as well …. medications will be a big thing but once you get into the mind frame, you're a qualified staff nurse we'll be fine”.*

Participant’s expressed a clear desire to travel now or soon, and their plans were affected by what they understood as requirements in other country’s.

*“Most places require experience, if you're applying for Australia, you need a year's experience in Ireland, I'm going to Australia to work I'd love to go after a few years”.*

For participant’s there was a lack of clarity regarding intellectual disability nursing in other country’s as only Ireland and the United Kingdom have a recognised division on the register.

*“It's not recognised in places like America …. there's some country’s I think you can work as a psychiatric or registered nurse”.*

Participant’s desire to travel was based on positive feedback from others and the fact that other friends had gone.

*“We've heard a lot of good feedback from older nurses that have gone and come back and there’s nurses that are gone and still out there, you'd hear feedback from there and from your friends”.*

The allure of travel was reinforced by what were perceived as a more positive work environment.

*“It's so positive like there's such a positive working environment, it's quite appealing”.*

However, some participant’s had to stay in Ireland due to family while other anticipated coming back in a few years. One participant intended to remain working abroad.

*“At the end of the day your family is still here, I'm a home bird, I want to go but I would come back, give me two, three years over there and then I would come back well I don't know even if I came home for Christmas”.*

## Discussion

The findings of this study indicate that further work is needed in; promoting intellectual disability nursing as a career choice, addressing professional esteem issues, support for educational and professional identity development during training programmes, providing career guidance opportunities prior to course completion and supporting the professional development of new graduates. In addition, while the findings of this study relate to intellectual disability, they may resonate across other nursing and midwifery disciplines and have implications for undergraduate and postgraduate education.

A key finding of this study was that some students choose intellectual disability nursing without knowing what it entails, had no experience to draw on and it was not their first career choice. In some instances, this resulted in students leaving the programme or transferring to another nursing programme. Consideration needs to be given to providing prospective students with opportunities to gain insight to the discipline of intellectual disability nursing through university open days, information on university websites and through social media. There is a need to rethink intellectual disability curricula to address the esteem issues raised by participant’s. Consideration needs to be given to facilitating nursing students in professional identity development through e.g., adjusting curricula content to include, the history of intellectual disability nursing, developing clinical skills and addressing identity and socialisation into the profession. Within this study participant’s identified their needs in terms of clinical skills development, and this should be considered in terms of future proofing the discipline of intellectual disability nursing [45, 46]. Professional identity is identified as the values and beliefs possessed by the nurse that guide his/her thinking, actions, and interactions with a patient [47]. Addressing professional identity may also address role ambiguity which often occurs when nursing students are unclear of their responsibilities or receive unclear information regarding the nursing student role. In this study some students felt that while they were on clinical placement they were fulfilling a care staff role rather than that of a student nurse focused on learning, development of skills and linking theory to practice and this finding may translate across nursing and midwifery programmes internationally as the focus can be on doing as opposed to the student learning opportunities and outcomes.

Through addressing professional identify issues nursing students can learn about the values and ethics of the profession, develop greater understanding of their role and their personal identification as a professional within an identified professional group [48]. Addressing a disciplines professional identify may address attrition rates and reduce turnover rate [49] and hold relevance across all areas of nursing and midwifery. In addition, other factors reported as contributing to attrition need to be considered such as; academic failure, personal/family difficulties, nursing as a wrong career choice, financial difficulties [50], disappointed dreams and lack of academic support [51, 52].

Because professional identity and the historical context of intellectual disability nursing are inextricably linked it is important to address this in the undergraduate curricula [53]. Intellectual disability nursing has operated on the fringes of nursing and has even been described as marginalised within the profession [21, 54–57]. Therefore, it is essential that intellectual disability nursing students develop a sense of belonging within the wider field of nursing, through the application of the unique knowledge, skills, values and culture of intellectual disability nursing while actively engaging in practice [58–60]. Furthermore, the professional identity may be eroded by the focus of governmental funding to service providers based on a social model and the lack of appreciation of how the social model can underpin intellectual disability nursing care and support delivery [25]. Developing a sense of belonging is synonymous with developing a sense of confidence and professionalism [61] and within practice students’ need to feel part of a team [62] rather than on the fringes, as clinical practice and learning experiences are linked to student satisfaction [63, 64]. A positive clinical learning environment fosters student progression and retention through facilitating their learning and identity as a nurse [65, 66] and reducing role uncertainty. Fundamental within this process it the recognition of the need for and value of intellectual disability nurses, where intellectual disability nurses are direct care experts, care coordinators, interprofessional collaborators, and advocates and leaders for equitable care [67]. Care is provided within a relationship-based, lifespan approach and intellectual disability nurses possess a unique relational skill set [68] characterized by a strong nurse-client relationship [69] that assists people with intellectual disability live and access healthy lives [70].

Undergraduate nurse education aims to develop competent, confident and effective professionals through education, socialisation and professional identity [34]. Developing professional identity is a core aspect of nursing that effects self-efficacy (confidence in accomplishing tasks), outcome expectation (benefits of working in the profession) [71] and professional commitment [72]. Poor socialisation can lead to negative professional identity [73], insufficient engagement [74] and hinder professional practice behaviour [75]. These aspects affect the ability to retain students upon course completion and influence their intention to leave [72, 73]. This loss of new graduate nurses is undesirable given the global demand for nurses [49] and nursing shortages [76]. Indeed, the recent World’s Nursing Report reveals a significant lack of nursing staff [77], likely to be even further exacerbated by the COVID-19 pandemic.

Within this study the professional identity of intellectual disability nursing was often questioned by participant’s and this maybe reflective of an exposure to or apathy observed by student nurses in practice, within existing registered intellectual disability nurses. This apathy in part may be due to registered nurses’ insecurity about their own professional identity, landscape changes within service provision and the ambiguity of their role created because of these changes [78]. However, lessons learned from COVID-19 highlight stress and burnout due to staffing shortages, the emotional toll of caring, illness, and feeling devalued [79]. Within intellectual disability this is further emphasised by the ambiguous role of intellectual disability nurses, deinstitutionalization, diverse practice settings, different models of care delivery and policies [22]. Thus, this has resulted in a cautious approach by disability providers to include nursing, fearing that intellectual disability nurses would medicalize the everyday lives of people with intellectual disability [80]. As a profession intellectual disability nurses need to make their contribution visible [7, 81] as they have been described as a shy discipline lacking a strong voice in articulating and explaining their role [82]. To progress and address this matter, both undergraduate and postgraduate educational programmes and the profession of intellectual disability nurses internationally need to address professional identify, the landscape of service provision models of care and professional advocacy.

A recent review of intellectual disability nursing in Ireland [6] articulates a clear role and focus for the intellectual disability nurse in Ireland and the timing is right to reinvigorate the profession and make its contribution visible [83]. Professional identity is formed through an interactive network and a student’s experience in the clinical learning environment may affect professional identity development [84]. Other factors, such as age, gender, educational background and family income have also been identified as contributing factors[85]. Nevertheless, it is through socialisation that a professional identity develops [86, 87] and both education and clinical placements are both major contributing factors in providing authentic learning and professional identity development opportunities [88, 89]. A key feature is the duration, quality and quantity of clinical placements which are linked to student satisfaction, their sense of belonging and professional identity [86, 90, 91]. Currently within the Irish nurse education system students undertake 63 weeks theoretical instruction and 81 weeks clinical instruction/internship and both elements are required to meet the nurse registration programmes standards and requirements [92] to be approved and accredited by the nursing and midwifery board of Ireland. The importance of nurses in clinical practice as role models during clinical placements and nurse academics as role models in the education setting cannot be underestimated as role modelling is an integral aspect of professional socialisation and occurs in all facets of the authentic learning journey [93]. In addition, research and collaboration across countries needs to be undertaken to identify and debate what is known about intellectual disability nursing internationally and beyond that of the country’s specifically providing direct entry training programmes (Ireland and the UK).

In this study participant’s were uncertain of career options and of opportunities for intellectual disability nursing in other country’s. This uncertainty of career options could be a factor influencing the retention of intellectual disability nurses and given that large numbers of students on the programme had prior experience and choose intellectual disability nursing there should be a positive attitude and a desire to remain in nursing related paid employment [94]. To further address the uncertainty within the discipline of intellectual disability nursing there needs to be a clear articulation of the implementation plan based on the outcomes of the national review of the future role of intellectual disabilities nurses in Ireland [6]. Furthermore, there is a need for a commitment from intellectual disability services within the national health service executive and outside (voluntary organisations) to the enactment of the implementation plan, continuous professional development of staff and advancement of the intellectual disability nursing profession. At an individual level intellectual disability nurses need to advocate for their role, articulate their practice and engage in on-going professional development and education. In this study participant’s expressed a desire for continuing professional education and career advancement, such desires are in line with national policy such as the policy on the development of graduate to advanced nursing and midwifery practice [95] and Sláintecare citizen care masterplan [96] which both support the effective use of intellectual disability nurses through service redesign based on population health planning, current levels of service delivery and the development of accessible healthcare services for people with intellectual disability.

Given that intellectual disability operates on the fringes of the nursing profession and that the size of the specialty of intellectual disability nursing accounts for just 6.75% of the nursing profession [97] there is a need for national leadership from within the profession, health service providers and health agency. Given the evidence of the complex health needs, access issues and health disparities of people with intellectual disability it is essential that we recruit and retain nurses with specialised unique knowledge and skills, and competence to work with people with intellectual disability [98–100].

This study highlights issues specific to intellectual disability nursing and as with any research the results have to be considered in the context of the study and limitations which include a small sample which was from one educational institute in Ireland. Additional research is required to identify the transferability of the findings and a longitudinal study within student training and post qualification is warranted given the anticipated workforce issues of recruitment, retention and existing workforce shortage. Previous research examining the work intentions of intellectual disability nursing students is limited and this paper adds to existing knowledge by providing a rich and detailed picture of personal, social and professional drivers.

## Conclusion

Intellectual disability service provision is in a time of major change for people with intellectual disabilities in Ireland [97]. This change and reconfiguring in the absence of a clear pathway for the intellectual disability nurse role within primary and community care teams creates confusion and role ambiguity. Growing numbers of social care workers are now recruited into the intellectual disability sector with demand outstriping supply [100], while important for care provision social care workers cannot replace the clinical care an intellectual disability nurse can provide. While there has been a national review of intellectual disability nursing which highlights the need for intellectual disability nurses [6], there is a vacuum which has been created in the absence of a visible and communicated plan for the professional work group. Such an absence seems to be creating an apathy and uncertainty regarding intellectual disability nursing. In addition, the absence of national identifiable intellectual disability nurse leaders and awareness of their role is affecting the level of undergraduate students’ entering and remaining in the intellectual disability nursing profession. Within the intellectual disability nursing students, the current ambiguity is affecting their future career choices and visions for nursing careers. Where some can see a pathway to advanced and community practice, others question the opportunities available and development of the profession that will occur if they stay within the profession.

## Data Availability

Data will not be shared due to difficulties in the anonymization of qualitative data as participant’s within this study shared their views and experiences with the assurance that their confidentiality and anonymity would be protected. Hence, the research data is not available publicly because this would compromise individual privacy and our ethical approval conditions. If you have a data request you may contact the lead author Dr Owen Doody at owen.doody@ul.ie.

## Abbreviations

BSc: Bachelor of Science
NHS: National Health Service (UK)
PIL: Participant Information Leaflet
UK: United Kingdom
WHO: World Health Organisation
